# Hidradenitis Suppurativa Patient Referrals to a Canadian Community Dermatology Practice: A Retrospective Chart Review

**DOI:** 10.1177/12034754231223451

**Published:** 2024-01-19

**Authors:** Leah A. Johnston, Susan M. Poelman

**Affiliations:** 1 Cumming School of Medicine, University of Calgary, Calgary, AB, Canada; 2 Division of Dermatology, University of Calgary, Calgary, AB, Canada; 3 Beacon Dermatology, Calgary, AB, Canada

**Keywords:** dermatology, hidradenitis suppurativa, referrals, interdisciplinary care, diagnostic accuracy, medical education

## Abstract

**Introduction:**

Awareness of hidradenitis suppurativa (HS) among non-dermatology healthcare providers is essential to facilitate prompt diagnosis, treatment, and referral to dermatology for further management.

**Objective:**

The purpose of this study was to analyze recent referrals to a Canadian community dermatology practice and compare the diagnostic concordance rates for HS between dermatologists and non-dermatologists.

**Methods:**

This study was a single-centre, retrospective chart review that was completed at Beacon Dermatology in Calgary, AB. Patients who were referred by a non-dermatologist for suspicion of HS and/or were diagnosed with HS by a dermatologist at Beacon Dermatology for the first time between May 2020 and May 2023 were included. Referral letters and dermatology clinic notes were analyzed to extract patient demographics, suspected pre-referral and post-referral diagnoses, and interim management plans that were initiated by the referring provider.

**Results:**

A total of 451 patient charts with suspected and/or confirmed HS were retrieved from the clinic database. The median wait time from referral to the first dermatology appointment was 9.1 weeks. The average duration of HS symptoms was 7.3 years. HS was suspected by the referring provider and confirmed by a dermatologist in 286 cases (63%). Preliminary management was initiated in 60% of mild and 66% of moderate-to-severe cases.

**Discussion:**

Given the prolonged time to diagnosis of HS, increasing awareness among healthcare providers is essential. Furthermore, this study highlighted the low implementation rates for evidence-based treatment options in preliminary management plans. Ultimately, this study demonstrates the need for increased interdisciplinary education on HS management in Canada.

## Introduction

Hidradenitis suppurativa (HS) is a chronic inflammatory skin disease that is estimated to affect nearly 4% of Canada’s population and has been historically underdiagnosed.^1^ A 2020 Canadian survey found that on average, patients with HS visited multiple healthcare providers over a 7-year time period before receiving a correct diagnosis of HS.^2^ Delayed diagnosis of HS can have a significant negative impact on patients, as an increased time to diagnosis has been correlated with disease progression, higher rates of systemic inflammatory comorbidities, and impairment in patients’ professional and social lives.^3^

Primary care providers are patients’ first point of contact with the healthcare system and their recognition of HS is essential to help facilitate early diagnosis and management. Patients with acute HS flare-ups may present to urgent care clinics and emergency departments and it is important for providers in these settings to understand the chronicity of HS in order to address the need for follow-up care, in addition to managing acute HS lesions. Additionally, specialist physicians may frequently provide care to HS patients in their clinical practices. Gastroenterologists and rheumatologists may oversee the care of comorbid conditions, including inflammatory bowel disease and inflammatory arthritis. Infectious disease specialists may consult on HS cases in both outpatient and hospital settings, especially when initiation of intravenous antibiotics such as ertapenem is needed, and gynecologists and general surgeons may receive referrals for common misdiagnoses of HS, such as Bartholin’s gland cysts, sexually transmitted infections, and pilonidal cysts.^4^ Plastic surgeons and urologists may also be involved in the surgical management of HS. Thus, awareness of HS in specialist physicians is also imperative to avoid misdiagnoses and initiate effective treatment plans.

Given the prolonged wait times to see a dermatologist in many areas of Canada, it is important that referring providers are knowledgeable in HS and are comfortable initiating preliminary management to help ameliorate patients’ symptoms while they are waiting to see a dermatologist.^5^ While previous studies have analyzed diagnostic accuracy and management of HS in emergency department settings, diagnostic accuracy and trends in first-line management of HS have not been explored in-depth in other healthcare settings.^6-9^ This study sought to characterize the current awareness of HS and knowledge of evidence-based preliminary management options in Canadian healthcare providers by analyzing patterns of referrals to a community dermatology practice.

## Methods

### Study Design

The purpose of this single-centre, retrospective chart review was to analyze recent referral patterns from non-dermatologists, as well as the pre-referral and post-referral diagnostic concordance rates for HS. Ethics approval for this study was obtained from the University of Calgary’s Conjoint Health Research Ethics Board.

### Participant Selection

Beacon Dermatology’s electronic medical record system (Healthquest) was searched using the key terms “HS” and “hidradenitis suppurativa,” which allowed for all patient charts mentioning HS in any clinical documentation (including both referral letters and dermatology clinic notes) to be compiled for screening. Patients who were referred for suspicion of HS and/or were diagnosed with HS for the first time by one of the dermatologists at Beacon Dermatology in Calgary, AB between May 2020 and May 2023 were included in this study. Referrals for transfer of care after a diagnosis of HS was previously established by a dermatologist at a different clinic and patients who were diagnosed with HS by a dermatologist at Beacon Dermatology for the first time prior to 2020 were excluded.

### Outcomes

The contents of the referral letters that were sent to the clinic by the referring provider were analyzed to extract the suspected pre-referral diagnoses and interim management plans that were initiated prior to patients’ first dermatology appointments. Additionally, information from patients’ first dermatology visits was extracted from clinic notes and included age, sex, total duration of HS symptoms, and Hurley stage at diagnosis.

### Statistical Analysis

Data from the patient charts were compiled and summarized using descriptive analysis. Frequencies and percentages were used to report categorical data and continuous variables were reported as means, median values, and/or ranges.

## Results

A total of 825 patient records were retrieved in the initial search of Beacon Dermatology’s clinic database. Of these records, 374 were excluded for the following reasons: 111 were duplicate records, 151 were patients referred for transfer of care from a dermatologist and/or referrals after a diagnosis of HS was previously established by a dermatologist, 80 were charts of patients seen prior to 2020, 28 did not have HS listed as a diagnostic consideration in either the referral letter or the clinic notes and were retrieved in error by the database search, and 4 charts did not have referral letters present in the records.

A total of 451 patient charts with suspected and/or confirmed HS in either the referral letters and/or the clinic notes from dermatologists at Beacon Dermatology met inclusion criteria. The mean age of patients with HS was 35.5 years (range: 11–87 years) and 83% (n = 373) of patients were females (Supplementary Table S1). The median wait time from electronic initiation of referral by the referring provider to patients’ first appointments with a dermatologist was 9.1 weeks. At the first dermatology visit, patients reported that they had experienced HS symptoms for an average of 7.3 years (n = 389). Mild (Hurley stage I) or mild-to-moderate (Hurley stage I-II) HS was diagnosed in 55% of patients (n = 246), while moderate (Hurley stage II), moderate-to-severe (Hurley stage II-III), or severe (Hurley stage III) HS was diagnosed in 45% of patients (n = 200).

Most patients (91.8%) were referred by a primary care provider. Other referring provider specialties included emergency medicine, gynecology, general surgery, rheumatology, gastroenterology, and infectious diseases. Out of these 451 patients, 449 received a diagnosis of HS from a dermatologist at Beacon Dermatology, while 2 patients were referred by their family physicians for suspected HS but received alternate diagnoses of dissecting cellulitis of the scalp and a solitary sebaceous cyst, respectively, from a dermatologist at Beacon Dermatology. While most patients (90.7%, n = 409/451) were referred for evaluation of suspected HS or one of its misdiagnoses, 9.3% (n = 42/451) were referred for an unrelated dermatologic condition (i.e., skin cancer) and HS was brought up by the patient as an additional concern.

HS was suspected by the referring provider and confirmed by a dermatologist in 286 cases (63.4%). Some (4.9%, n = 22/451) referral letters did not specify a preliminary diagnosis but included terminology that may be used to describe HS, such as “recurrent painful boils.” In 22% of cases (n = 99/451), an alternate diagnosis was suspected by the referring provider and/or suspicion of HS was not mentioned. These patients were subsequently diagnosed with HS by their dermatologist. Misdiagnoses included abscesses, acne, contact dermatitis, sebaceous or epidermoid cysts, ingrown hairs, folliculitis, furuncles or carbuncles, pilonidal cysts, prurigo nodularis, sexually transmitted infections, skin infections, and skin cancer (Supplementary Figure S1). Preliminary management for HS was initiated in 63% (n = 256/407) of HS cases (Supplementary Table S1). A large proportion of patients (37%, n = 151/407) were not started on any treatment by their referring provider.

Subgroup analysis of mild HS (Hurley stage I or I-II) versus moderate-to-severe HS (Hurley stage II, II-III, or III) found that patients with mild HS had a 62% (n = 133/216) diagnostic concordance rate between non-dermatologists and dermatologists, while the diagnostic concordance in moderate-to-severe cases was 80% (n = 151/188) (Supplementary Figure S2). The diagnostic concordance rate between referrals from primary care providers (including family physicians and nurse practitioners) and diagnoses made by dermatologists was 74% (n = 278/375) and between other specialist physicians and dermatologists, the diagnostic concordance was 88% (n = 30/34). The rates of initiation of interim treatment by the referring provider for mild and moderate-to-severe HS were 60% (n = 129/216) and 66% (n = 124/188), respectively (Supplementary Figure S3a). Topical antibiotics were the most frequently prescribed treatment for both the mild and moderate-to-severe (n = 107/404, 26%) HS patient groups (Supplementary Figure S3b). Longer than 1-month courses of either oral tetracycline antibiotics or oral clindamycin with or without rifampin were prescribed in 8% of all cases (n = 33/404). Incision and drainage was the most frequently performed procedural intervention (n = 48/404, 12%), while intralesional corticosteroid injections and local in-office surgical excision were done in 2% (n = 9/404) and 1% (n = 3/404) of cases, respectively.

## Discussion

The results from this study provide valuable information regarding the current trends for HS patient referrals in Canada. Similarly to the 2020 Canadian Skin Patient Alliance’s HS Patient Experience survey, the average time to diagnosis of HS from the onset of HS symptoms in this study was around 7 years.^2^ The unchanged long delay in time to diagnosis suggests that there remains a need for increased awareness of HS in Canadian healthcare providers. In this study, the number of different providers seen for HS before receiving a diagnosis could not be obtained due to limitations in data from referral letters and Beacon Dermatology’s electronic medical record system. However, a previous Canadian survey found that 61% of HS patients visited 3 or more healthcare providers before receiving an accurate diagnosis.^2^ Although the pre-referral and post-referral diagnostic concordance rates for HS in this study were 74% and 88% in primary care providers and specialist physicians, respectively, the 7.3-year time to diagnosis suggests that the referring providers in this study were likely not the first healthcare providers that those patients had consulted for their HS symptoms. A potential explanation for the delayed time to recognition of HS is insufficient dermatology teaching in the general curriculum of many Canadian medical schools and limited dermatology clinical training in non-dermatology residency programs.^10,11^ In addition, a previous study found that HS was one of the least familiar dermatologic conditions among a group of American medical students, indicating that there is a need for increased teaching on HS during medical school.^12^ Among family physicians, a recent survey in Turkey demonstrated that while more than 90% were aware of HS as a diagnostic consideration, only 23.7% felt comfortable diagnosing HS in their clinical practices.^13^ These studies suggest that there is a need for research on the learning needs of medical students and primary care providers in Canada with respect to diagnosing and managing HS, in order to optimize their training in this area.

Many patients who were eventually diagnosed with HS by a dermatologist were diagnosed with conditions that can mimic some signs and symptoms of HS, including solitary sebaceous or epidermoid cysts, folliculitis, furunculosis, and carbuncles. The results of this study indicated that patients with mild HS were more likely to be misdiagnosed compared to patients with moderate-to-severe disease. It can be more challenging to distinguish early stage HS from other conditions due to a lack of characteristic lesions that are seen in advanced stages, such as sinus tracts. To diagnose HS, 3 criteria must be met: (1) Typical lesion morphology (double open comedones, papules, nodules, abscesses, sinus tracts, and/or scarring), (2) lesions occurring in typical locations for HS (especially the intertriginous areas), (3) chronicity and recurrence (2 or more characteristic lesions appearing during a time period of ≥6 months).^14^ If all 3 diagnostic criteria are met, there is a 90% sensitivity and a 97% specificity for a correct diagnosis of HS.^14^

Many patients (63%) who were referred for either HS or an alternate suspected diagnosis were started on one or more initial treatments by their referring provider. Hurley stage II and III patients were only slightly more likely (66%) than patients with mild disease (60%) to have been started on one or more therapies. For both severity groups, topical antibiotics were the most frequently prescribed therapy. While topical antibiotics may be considered as a first-line therapy for Hurley stage I HS, clinical practice guidelines suggest that topical therapies alone are often insufficient for managing Hurley stage II and stage III disease.^15^ In these patients, the use of systemic therapies, including oral antibiotics, antiandrogens, and/or biologic medications, is often necessary to provide effective disease control.

Oral antibiotics are often recommended as a first-line therapy for HS due to their anti-inflammatory effects.^15^ A 3-month course is typically needed to obtain a reduction in disease activity, and specific oral antibiotics that are prescribed for this purpose include tetracyclines and clindamycin, with or without rifampin.^15^ Additionally, treatment with oral antibiotics for up to 3 months is frequently required by Canadian health insurance companies prior to patients meeting qualifications for insurance coverage of biologic therapies, which have the strongest supporting evidence from the literature for the treatment of moderate-to-severe HS.^15^ Only a small percentage of patients with moderate-to-severe HS (11%, n = 21/188) in this study were started on a prolonged (≥1 month) course of either oral tetracycline antibiotics or oral clindamycin with or without rifampicin. Wait times to see a dermatologist for HS at Beacon Dermatology are approximately 9 weeks, which is shorter than the national median wait time for dermatology referrals in Canada, which is about 13 weeks.^16^ During this time, initiation of oral antibiotic treatment by patients’ referring providers is recommended to provide effective interim management and reduce delays to initiation of biologic therapies, if needed.

Nearly 12% of all patients that were referred for HS underwent an incision and drainage procedure with their referring provider. While incision and drainage can be useful for acute relief of pain from HS flare-ups, it has a high recurrence rate and is not the preferred procedural intervention for HS.^17^ Intralesional corticosteroid injections and local surgical deroofing, which is an office-based procedure that involves removal of damaged tissue from HS lesions and sinus tracts, have stronger supporting evidence for their use in managing acute HS flare-ups.^15,17^ Additionally, surgical deroofing has the lowest rate of recurrence of the different surgical interventions for HS.^17^ However, only a small number of patients in this study underwent treatment with intralesional corticosteroids (2%) and local surgical excision (1%). At Beacon Dermatology, the clinic’s policy offers urgent fit-in appointments for HS patients with acute flare-ups to receive intralesional corticosteroid injections. Patients with recurrent HS lesions and sinus tracts are managed with deroofing surgery. Interim management of acute HS flares with either intralesional corticosteroid injections or deroofing surgery (depending on provider experience) is advised to help alleviate patients’ pain while they are waiting to receive further management with a dermatologist. Additionally, training in surgical deroofing may be useful for providers who practice in urgent care clinics or emergency departments, as an alternative to incision and drainage procedures. A summary of recommendations for preliminary management of HS is provided in Supplementary Table S2.

A limitation of this study is the potential for sampling bias as a confounder of diagnostic concordance rates and preliminary management data. Providers in this study who elected to refer their patients to a dermatologist may have higher levels of awareness of HS compared to other healthcare providers within the same geographic region and may also be more knowledgeable of the treatment options for HS patients, some of which require prescription by a dermatologist. Another potential limitation is that this study relied on retrospective data collection from referral letters and clinic notes. While a standardized note template is used in clinic notes from new HS patient consultations at Beacon Dermatology, the level of detail in the referral letters was highly variable, which may have led to incomplete reporting of some aspects of patients’ medical histories, including previous HS treatments.

In summary, this study highlights some of the current gaps in care for HS patients in Canada. Given the prolonged time to diagnosis, increasing awareness of HS among healthcare providers is an important goal. Recognizing potential signs of HS and understanding how HS can be differentiated from other conditions with overlapping symptoms is a particularly important skill to focus on for primary care providers and specialists who routinely treat HS patients. Furthermore, this study demonstrates the current gaps in preliminary management for HS patients, including low implementation rates for evidence-based treatment plans. Increasing referring providers’ understanding of HS management is especially important for patients with Hurley stage II and III HS and for patients who present with acute or recurrent HS flare-ups. Ultimately, the results of this study suggest that there is a need for increased teaching about HS in medical schools, as well as development of continuing medical education courses on HS management for practicing providers, to help optimize interdisciplinary care for HS patients.

## Supplemental Material

sj-docx-1-cms-10.1177_12034754231223451 – Supplemental material for Hidradenitis Suppurativa Patient Referrals to a Canadian Community Dermatology Practice: A Retrospective Chart Review Supplemental material, sj-docx-1-cms-10.1177_12034754231223451 for Hidradenitis Suppurativa Patient Referrals to a Canadian Community Dermatology Practice: A Retrospective Chart Review by Leah A. Johnston and Susan M. Poelman in Journal of Cutaneous Medicine and Surgery
