# Supplementary material for: Hidradenitis Suppurativa Patient Referrals to a Canadian Community Dermatology Practice: A Retrospective Chart Review
Source: J Cutan Med Surg. 2024 Jan 19;28(2):136–40. doi: 10.1177/12034754231223451 (PMC11015712; doi:10.1177/12034754231223451)
Supplement: sj-docx-1-cms-10.1177_12034754231223451 – Supplemental material for Hidradenitis Suppurativa Patient Referrals to a Canadian Community Dermatology Practice: A Retrospective Chart Review [file sj-docx-1-cms-10.1177_12034754231223451.docx]

**Supplemental Material**

**Table S1.** Patient Demographics, Clinical Features of Hidradenitis Suppurativa and Characteristics of Referrals to Dermatology.

| **Characteristic** | **Data Category** | **Frequency (%) or Numerical Data** |
| --- | --- | --- |
| Sex (n=449) | Females | 373 (83%) |
|  | Males | 76 (17%) |
| Age (n=449) | Mean | 35.5 years |
|  | Median | 35 years |
|  | Range | 11-87 years |
| Wait Time from Referral to Dermatology Appointment (n=409)^a^ | Median | 9.1 weeks |
|  | Range | 1-258 days^b^ |
| Total Duration of HS Symptoms at First Visit (n=389)^c^ | Mean | 7.3 years |
|  | Median | 4 years |
|  | Range | 0.1-50 years |
| Hurley Stage of HS (n=446) | Stage I | 232 (52%) |
|  | Stage I-II | 14 (3%) |
|  | Stage II | 175 (39%) |
|  | Stage II-III | 8 (2%) |
|  | Stage III | 17 (4%) |
| Referring Provider Specialty (n=451) | Nurse Practitioner (Primary Care) | 5 |
|  | Family Medicine Physician | 409 |
|  | Emergency Medicine | 14 |
|  | Gastroenterology | 3 |
|  | Gynecology | 10 |
|  | Rheumatology | 4 |
|  | General Surgery | 5 |
|  | Infectious Diseases | 1 |
| HS Suspected by Referring Provider? (n=451) | Yes—HS subsequently confirmed by dermatologist | 286 (63.4%) |
|  | Yes—Diagnosis of HS excluded by dermatologist | 2 (0.4%) |
|  | No—Alternate diagnosis suspected | 99 (22.0%) |
|  | No suspected diagnosis specified in referral letter, but features of HS (i.e. “recurrent painful boils”) were described | 22 (4.9%) |
|  | Not applicable—HS was not the primary dermatologic indication for referral | 42 (9.3%) |
| Preliminary Management Initiated by Referring Provider? (n=407)^a^ | Yes^d^ | 256 (63%) |
|  | No | 151 (37%) |

HS, hidradenitis suppurativa.

^a^ Excludes patients who were referred for an unrelated dermatologic indication (i.e. skin cancer) and HS was discussed as an additional concern during the dermatology consultation.

^b^ Excludes patients who did not report a specific, quantitative duration of HS symptoms.

^c^ The range does not account for rescheduled dermatology consultations, as there were some patients who were initially assigned an earlier appointment date but subsequently cancelled and rescheduled their appointments.

^d^ Includes patients who were started on treatment with tumor necrosis factor-α inhibitor biologics by the referring provider for pre-existing inflammatory bowel disease or rheumatologic indications (n=5).

**Table S2.** Recommendations for First-Line Management of HS by Hurley Stage.

| **Hurley Stage I** | **Hurley Stage II-III** |
| --- | --- |
| - Topical clindamycin 1-2% - Topical resorcinol cream 15% | - Oral tetracycline antibiotics (doxycycline 100 mg OD-BID or minocycline 100 mg QHS x 12 weeks) - Oral clindamycin (300 mg BID) & rifampin (300 mg BID) x 12 weeks |
| **All Stages** | |
| - Counselling on potential triggers for HS: smoking, menstrual cycle hormonal fluctuations, skin friction, excessive sweating, shaving or waxing affected areas, being overweight or obese, and diet (high consumption of refined sugars and carbohydrates, skim milk products and whey protein shakes). - Provide support for smoking cessation, weight loss, and/or dietary modifications. - Female patients: consider antiandrogenic hormonal therapies, such as oral contraceptive pills containing drospirenone or cyproterone acetate, or spironolactone (100-200 mg daily). - Intralesional triamcinolone acetonide injections (10-20 mg/cc x average 0.5-1cc) for acutely inflamed lesions. - Depending on provider training and comfort, deroofing surgery may be considered for individual recurrent HS lesions and sinus tracts. | |

**Supplemental Figure Legends**

**
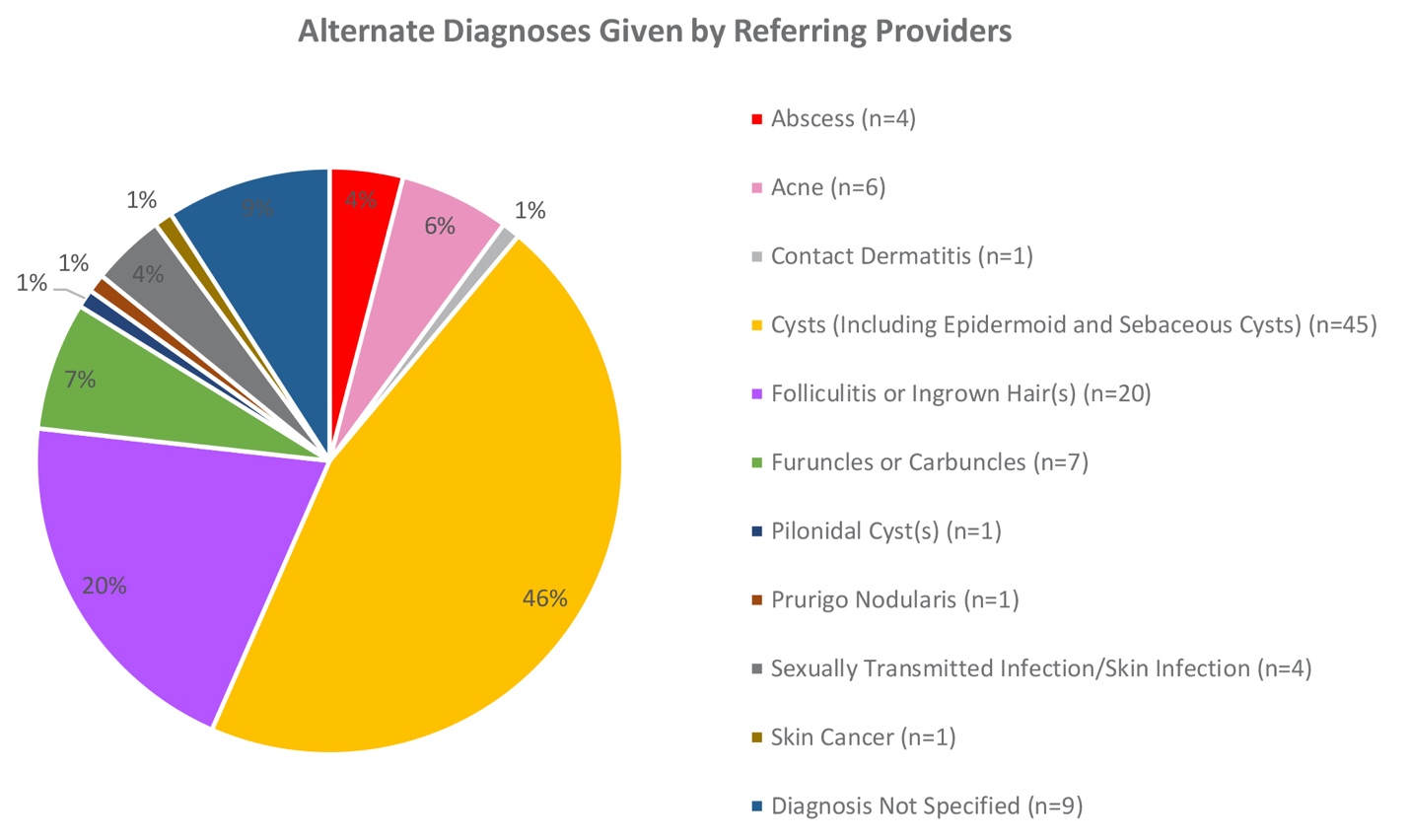
**

**Figure S1.** Alternate diagnoses provided by referring providers in patients who were subsequently diagnosed with hidradenitis suppurativa by a dermatologist.

**
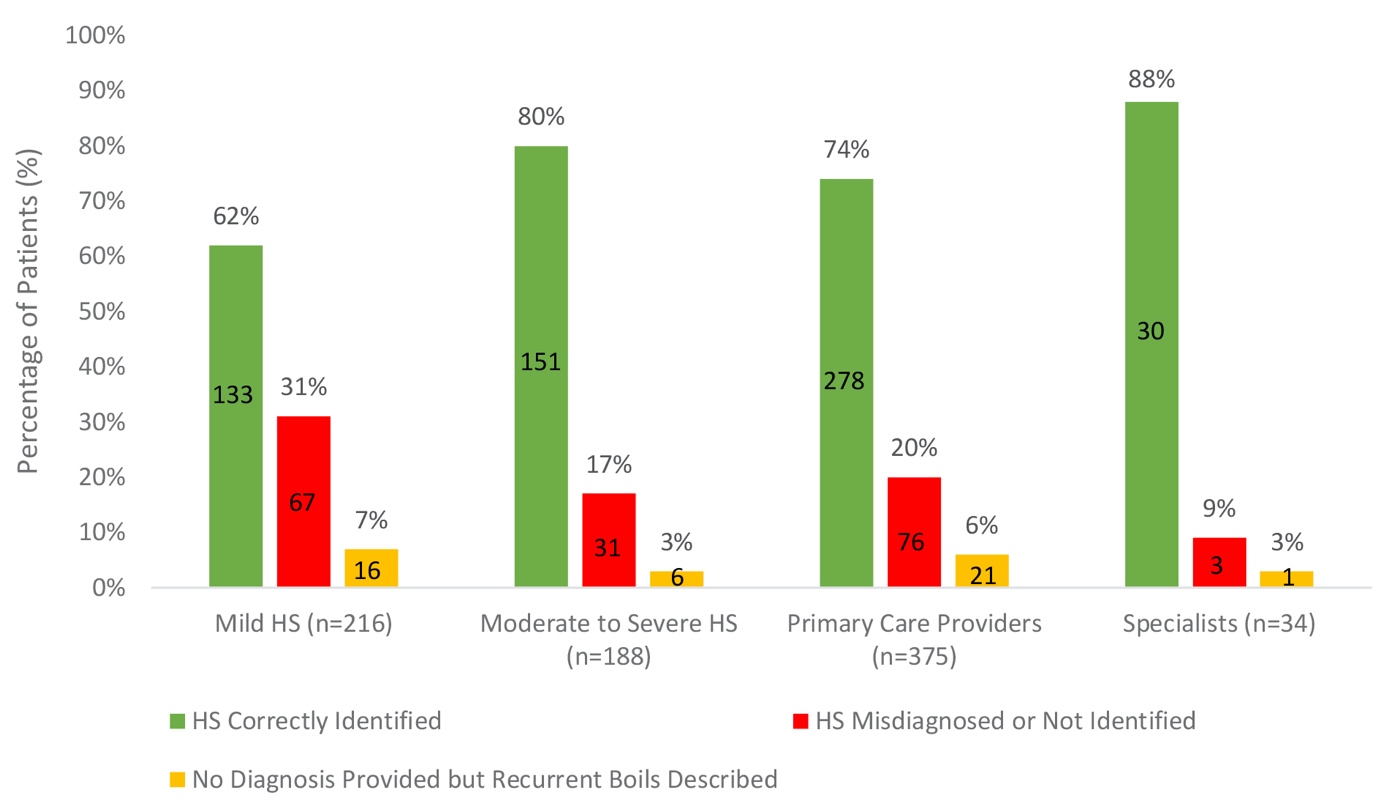
**

**Figure S2.** Diagnostic concordance rates between non-dermatology healthcare providers and dermatologists for hidradenitis suppurativa (HS), stratified by disease severity and referring provider specialty.

*Data excludes patients who were referred for unrelated dermatologic presentations.

**
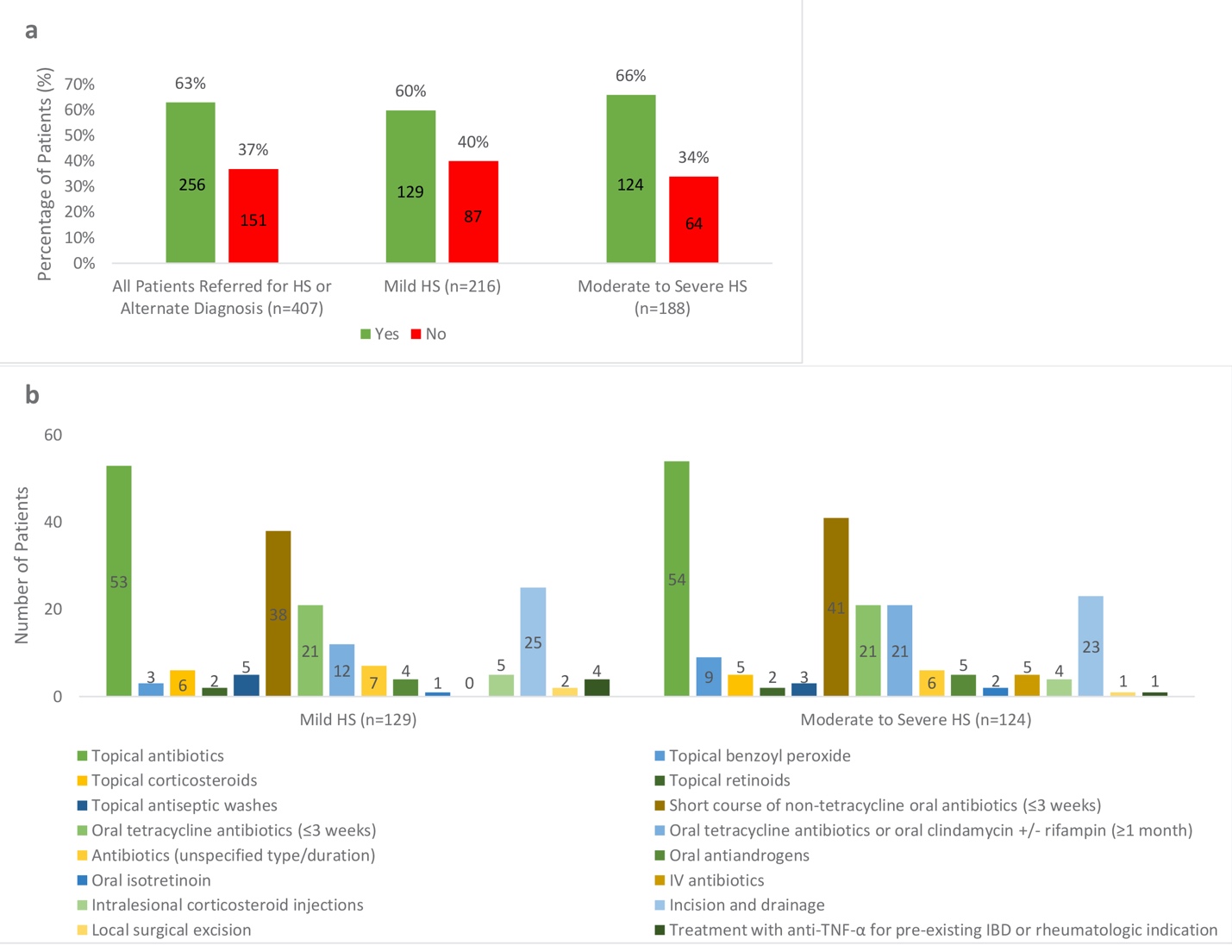
Figure S3a.** Rates of preliminary management initiation in patients that were subsequently diagnosed with hidradenitis suppurativa (HS) by a dermatologist, stratified by severity of HS.

**Figure 3b.** Types of treatments initiated by referring providers.
